# Supplementary material for: Superoxide Dismutase-1 Intracellular Content in T Lymphocytes Associates with Increased Regulatory T Cell Level in Multiple Sclerosis Subjects Undergoing Immune-Modulating Treatment
Source: Antioxidants (Basel). 2021 Dec 3;10(12):1940. doi: 10.3390/antiox10121940 (PMC8750574; doi:10.3390/antiox10121940)
Supplement: Supplementary file 1 [file antioxidants-10-01940-s001.zip › antioxidants-1459422-supplementary.pdf]

Supplementary Table S1 Patient clinical features, according to the treatment they underwent

Treatment	Sex N (%)	EDSS score
Fingolimod	F=7 (58)	1.7±0.8
Teriflunomide	F=9 (60)	1.8±0.9
Glatiramer-acetate	F=10 (63)	2.23±1.07
IFNb-1b	F=10 (71)	1.73±0.83
Dimethyl Fumarate	F=11 (69)	1.77±0.87
Clabridine	F=4 (80)	1.25±0.25
